# Verification of a novel point-of-care HbA_1c_ device in real world clinical practice by comparison to three high performance liquid chromatography instruments

**DOI:** 10.11613/BM.2018.020705

**Published:** 2018-04-15

**Authors:** Yufei Wang, Wei Peng, Junling Tang, Lu Dong, Chengchen Gu, Xin Zhang, Jian Zhou, Weiping Jia

**Affiliations:** 1 Shanghai Diabetes Institute, Shanghai Jiao Tong University Affiliated Sixth People’s Hospital; Shanghai Key Laboratory of Diabetes Mellitus, Shanghai, China; 2 Wuxi Biohermes Bio&Medical Technology Co., Ltd., Wuxi, China; 3 Department of Endocrinology and Metabolism, Shanghai Jiao Tong University Affiliated Sixth People’s Hospital, Shanghai Clinical Center for Diabetes, Shanghai Key Clinical Center for Metabolic Disease, Shanghai, China

**Keywords:** point-of-care testing, glycated haemoglobin A1c, diabetes mellitus, evaluation studies

## Abstract

**Introduction:**

A real world clinical study was designed and conducted to evaluate the performance of a novel point-of-care device for determination of glycated haemoglobin A_1c_ (HbA_1c_), A1C EZ 2.0, in daily clinical practice.

**Materials and methods:**

Five hundred and fourteen subjects were included in this study, and divided into three groups. HbA_1c_ was measured by A1C EZ 2.0 and three different high performance liquid chromatography (HPLC) devices: Bio-Rad Variant II Turbo, Tosoh HLC-723 G8 and Premier Hb9210 separately. Precision of A1C EZ 2.0 was also evaluated.

**Results:**

Results obtained from A1C EZ 2.0 and all HPLC devices are correlated. Passing-Bablok regression analysis shows the equation of A1C EZ 2.0 results against the mean of HPLC devices with corresponding 95% confidence intervals (95% CI) for the intercept and slope is y = 0.10 (- 0.17 to 0.10) + 1.00 (1.00 to 1.04) x. Bland-Altman difference plot shows that the mean relative difference between A1C EZ 2.0 and Variant II Turbo, G8, Hb9210 and all HPLC results is 2.5%, 0.6%, 0.4% and 1.1%, respectively. In addition, 121 pairs of results determined by using both venous and capillary blood prove that the difference of two kinds of blood sample causes no notable variation when measured by A1C EZ 2.0. Precision study gives 2.3% and 1.9% of total coefficient of variation for normal and abnormal HbA_1c_ sample in A1C EZ 2.0.

**Conclusions:**

HbA_1c_ values measured by A1C EZ 2.0 were in good accordance with the results obtained with the reference HPLC devices.

## Introduction

Diabetes mellitus (DM) has become a cause of serious concern worldwide, as well as in China. The prevalence of DM and pre-diabetes among adult Chinese population in 2010 was estimated to be 11.6% and 50.1%, respectively, representing 113.9 and 493.4 million people. However, only 28.4% of DM patients have been diagnosed till now in China (*1*). This implies that over eighty million patients with undiagnosed DM still need to be screened and diagnosed, which can be a major challenge for the public health system of China. Glycated haemoglobin A_1c_ (HbA_1c_) values, which can reflect the average blood glucose concentration over the past 90-120 days, is now not only a standard index for evaluating the effectiveness of treatment, but also a widely acknowledged parameter for screening and diagnosing DM (*2*-*4*). High performance liquid chromatography (HPLC) is currently widely used for HbA_1c_ measurement in clinical practice, but it is generally used in the hospital central laboratory, where physician and patients have to wait for 1-3 days for getting the test results. Point-of-care (POC) HbA_1c_ devices, which can provide test results within few minutes, is fast becoming a tool for the screening and diagnosis of DM at a relatively lower cost. Clinical studies have shown that POC devices help doctors in making timely adjustments in treatment plan, which further brings additional benefits to long-term glycaemic control (*5*-*7*). On the other hand, there were common concerns about the accuracy and precision of POC HbA_1c_ devices. Lenters-Westra *et al.* have studied the performance of eight POC HbA_1c_ devices in 2009, and only two of them met the generally accepted performance criteria (*8*). Most of POC HbA_1c_ devices in the study failed to achieve the total CV of 3%, and show notable differences of analytical performance between different reagent lots. A similar investigation of seven POC HbA_1c_ devices has been performed by the same authors in 2014 (*9*). Generally, significant improvement has been found in precision and accuracy of these devices, four of seven devices met the generally accepted performance criteria, though some specific problems, such as interferences or choice of reference device for calibration has been found in this study (*9*).

Here, we performed systematic evaluation of a novel hand-held HbA_1c_ POC device, A1C EZ 2.0 (Biohermes, Wuxi, China; measurement range 4~14% HbA_1c_) in a clinical study by comparison to HPLC instruments. Zhou *et al.* reported a study of A1C EZ 2.0 when our investigation was in progress, and their results show this device has favourable performances (*10*). In order to minimize the potential random bias caused by a definite reference device, three HPLC instruments were involved in our study, and their mean result for each sample, as well as test results by individual HPLC instruments, were used as reference values for comparison. Linear regression analysis, bias plot, as well as sensitivity and specificity analysis of A1C EZ 2.0 were performed based on the data obtained in the comparison study. In addition, the precision of A1C EZ 2.0 was also evaluated in this study. All instruments used in this study were certified by both the National Glycohemoglobin Standardization Program (NGSP) and the International Federation of Clinical Chemistry and Laboratory Medicine (IFCC) in 2016.

## Materials and methods

### Study design

We performed an analytical verification study of the POC HbA_1c_ device A1C EZ 2.0 by comparison with three other IFCC certified secondary reference method (SRM) HPLC instruments. Blood samples were collected from subjects in hospital, and the study lasted for seven months (detail information of instruments and subjects are given below).

Both venous and fingertip blood samples were used in the comparison, to investigate the test results difference between A1C EZ 2.0 and three HPLC instruments. Potential difference of test results caused by sample types was also studied by testing both venous and fingertip blood from the same subjects in the same instrument in this study.

We also evaluated the precision of A1C EZ 2.0 following CLSI EP5-A2 (Evaluation Of Precision Performance Of Quantitative Measurement Methods. Approved Guideline - Second Edition). Two potassium-ethylenediaminetetraacetic acid (K_2_-EDTA) anticoagulant whole blood samples (one within normal range and the other in the higher range of HbA_1c_ values) were tested in two runs per day in duplicate on twenty consecutive days. Controls provided by manufacturers were used before and after every day’s experiments to ensure that performance of all instruments meets the required standards by manufacturers. Otherwise calibration was carried out following manufacturers’ manual.

### Subjects

After approval from the institutional review committee of Shanghai Jiao Tong University Affiliated Sixth People's Hospital (Shanghai, China), and in accordance with the principles of the Helsinki Declaration II, we randomly recruited 514 subjects from the Outpatient Departments of the Endocrinology and Metabolism of Shanghai Jiao Tong University Affiliated Sixth People's Hospital. We included both diabetes and non-diabetes cases aged fourteen and older. We excluded specimens from subjects with anaemia, haemolytic disease, red cell cycle abnormalities, nephropathy and other known comorbidities, whose diseases were reported through a questionnaire survey or determined by relevant laboratory results. Subjects aged 60 (15 - 93) were enrolled from April to October 2016. Median age of male and female subjects was 61 and 60, respectively.

All subjects have signed informed consent before their blood sample being collected. Subjects were divided into three groups, venous blood samples were collected from 197 subjects (subject group I, SGI), fingertip capillary whole blood samples were collected from other 196 subjects (subject group II, SGII), and both venous blood samples and fingertip capillary whole blood samples were collected from the rest 121 subjects (subject group III, SGIII).

### Sample collection

All venous blood samples were collected in the standard tube (3mL, WeiGao Group, China) containing K_2_-EDTA anticoagulant, stored at 4 °C and then tested within 24 hours of collection. One tube of venous blood sample was collected from each subject in SG I and SG III. For subjects in SG II, the fingertip capillary whole blood samples were directly used for the tests by A1C EZ 2.0, fingertip capillary whole blood samples were also collected in one mini tube containing K_2_-EDTA anticoagulant and tested with three the HPLC devices within 24 hours.

### Methods

The HbA_1c_ values from each blood sample were measured by A1C EZ 2.0 and all three HPLC devices: Bio-Rad Variant II Turbo (Bio-Rad Laboratories, Hercules, USA; measurement range 3.5 - 19% HbA_1c_), Tosoh HLC-723 G8 (Tosoh, Tokyo, Japan; measurement range 4 - 16.9% HbA_1c_) and Premier Hb9210 (Trinity Biotech, Bray, Ireland; measurement range 3.7 - 18.5% HbA_1c_). Both A1C EZ 2.0 and Premier Hb9210 utilize boronate affinity method; however, A1C EZ 2.0 uses lateral chromatography on a porous membrane matrix, while Premier Hb9210 HPLC device uses column chromatography. Tosoh HLC-723 G8 and Bio-Rad Variant II Turbo devices utilize cation-exchange method. All tests were performed according to the manufacturers’ instructions manual. Operators performing tests on the A1C EZ 2.0 and HPLC devices were blinded to the clinical characteristics of the subjects.

### Statistical analysis

#### Linear regression analysis

Passing-Bablok linear regression analysis of HbA_1c_ values obtained by A1C EZ 2.0 versus three HPLCs was further conducted to assess the accuracy of A1C EZ 2.0 while considering the above mentioned HPLC devices as references (*11*). The linear regression analysis in each subject group was performed separately. Six hundred and thirty-five pairs of data from all 514 subjects (121 subjects tested both venous blood and fingertip capillary whole blood) were also combined for linear regression analysis for each reference HPLC device. Finally, to check overall agreement independent of chosen reference HPLC device, the mean HbA_1c_ values of three HPLC devices (referred as “mean SRM” below) were used to perform the linear regression against results given by A1C EZ 2.0.

#### Bias plot, absolute value of relative difference and mean absolute value of relative difference

Bland-Altman difference plot was performed to evaluate the difference between the HbA_1c_ values given by A1C EZ 2.0 and HPLC devices (*12*). Mean difference as well as the mean relative difference between A1C EZ 2.0 results and each HPLC results were calculated. The limits of agreement for the difference between A1C EZ 2.0 and each HPLC device were also calculated to reflect the distribution of these differences. In addition, the absolute value of relative difference (ARD) analysis and mean absolute value of relative difference (MARD) analysis was performed. Absolute value of relative difference and MARD were calculated by using the following equations:

ARD = (|A1C EZ 2.0 value – HPLC value| / HPLC value) × 100%

MARD = Average (ARD).

The distribution of the difference was further analysed based on the data of ARD and MARD. Six hundred and thirty-five pairs of data from all 514 subjects were combined and used for bias plot, ARD and MARD analysis.

#### Precision

Precision of A1C EZ 2.0 was evaluated using CLSI EP5-A2. The calculation of within-run, between-run, within-day and total coefficient of variance was followed by the standard method provided in CLSI EP5-A2.

#### Comparison of venous blood results and fingertip capillary whole blood results

Values of 121 pairs of venous blood and fingertip capillary whole blood on A1C EZ 2.0 were compared by Passing-Bablok linear regression analysis, to see if there is a significant difference between the two kinds of blood samples.

#### Sensitivity and specificity analysis

Sensitivity and specificity were calculated by using HbA_1c_ clinical decision limit for DM diagnosis of 6.5% and mean SRM or Premier Hb9210 as reference value. MedCalc (Ostend, Belgium) statistical software (version 16.4) was used to perform statistical analyses.

## Results

### Overall results

A total of 514 subjects, including 266 men and 248 women, were randomly recruited in this study. The venous and fingertip capillary whole blood were tested in the SGI (N = 197) and SGII (N = 196) group, respectively. SGIII (N = 121) subjects had evaluations of both venous blood and fingertip capillary whole blood.

**Figure 1:**
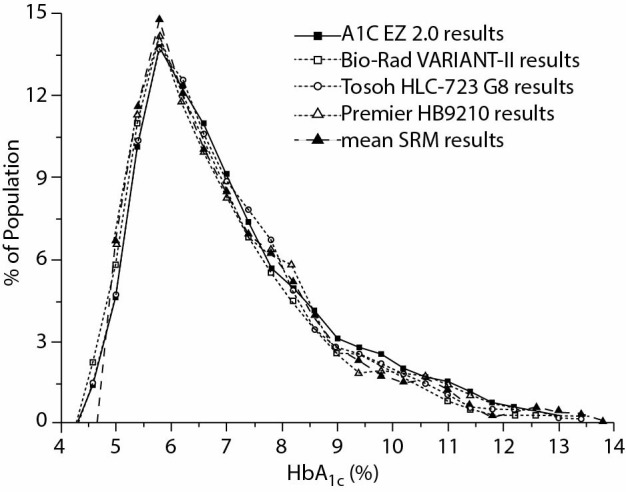
Frequency distribution of the HbA_1c_ values measured by the A1C EZ 2.0 and three secondary reference methods based on HPLC methodology including Bio-Rad Variant II Turbo, Tosoh HLC-723 G8 and Premier Hb9210. Distribution of mean SRM is also included in the figure. In total, 635 samples were analyzed by each instrument. The population percentage (y-axis) is plotted against HbA_1c_ values (x-axis).

The frequency distribution patterns of HbA_1c_ values for all four instruments were similar (Figure 1). HbA_1c_ values of all subjects ranged from 4.4 - 13.0% for A1C EZ 2.0 and from 4.5 - 13.6% for mean SRM, *i.e.* mean of all three HPLC devices. Median (95% confidence intervals, CIs) HbA_1c_ of A1C EZ 2.0 was 6.9% (6.7 - 7.0), and 6.7% (6.6 - 6.9) for all three HPLC devices put together (mean SRM) (Table 1).

**Table 1:** HbA_1c_ results for all the devices investigated

	**A1C EZ 2.0**	**Bio-Rad VARIANT-II**	**Tosoh HLC-723 G8**	**Premier HB9210**	**Mean SRM**
HbA1c range (%)	4.4 - 13.0	4.4 - 13.2	4.4 - 13.3	4.7 - 14.3	4.5 - 13.6
Median (95% CI)	6.9 (6.7 - 7.0)	6.7 (6.5 - 6.9)	6.8 (6.6 - 7.0)	6.8 (6.6 - 6.9)	6.7 (6.6 - 6.9)
KS test	P < 0.001	P < 0.001	P < 0.001	P < 0.001	P < 0.001
Skewness coefficients	1.062 (P < 0.001)	1.190 (P < 0.001)	1.199 (P < 0.001)	1.282 (P < 0.001)	1.226 (P < 0.001)
Kurtosis coefficients	0.678 (P = 0.011)	1.312 (P < 0.001)	1.348 (P < 0.001)	1.539 (P < 0.001)	1.388 (P < 0.001)
KS test - Kolmogorov-Smirnov test for normal distribution. Skewness coefficients and Kurtosis coefficients were used for normal distribution determination. SRM - secondary reference method.					

### Linear regression

**Figure 2:**
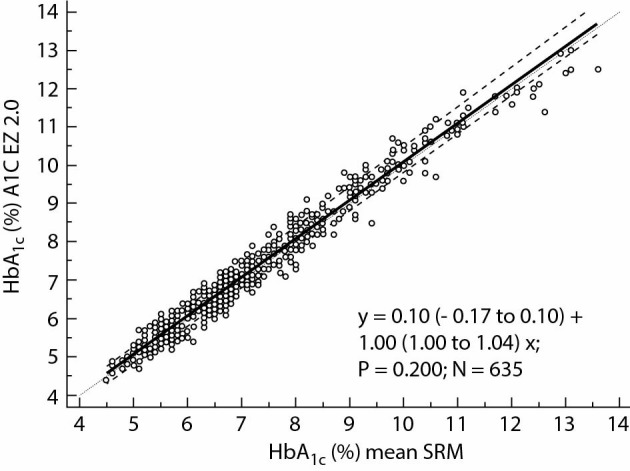
Scatter diagram and linear regression analysis of A1C EZ 2.0 results *versus* mean SRM. The regression line (solid line), 95% CIs (dashed lines) and the identity line (y = x, dotted line) are displayed. SRM- secondary reference method.

Linear regression analysis indicates that test results of A1C EZ 2.0 show good agreement with the results obtained by reference HPLC devices (Table 2). The linear regression equation of A1C EZ 2.0 values against mean SRM is presented in Figure 2. A1C EZ 2.0 is most closely related to Premier Hb9210 of all the three reference HPLC devices. The slope and intercept given by linear regression between A1C EZ 2.0 and Premier Hb9210 with all of the combined data is 1.00 (1.00 to 1.00) and 0.00 (- 0.00 to 0.00), respectively, which coincides with the ideal y = x function.

**Table 2:** Regression analysis of A1C EZ 2.0 results *versus* three secondary reference methods and the mean SRM

**Comparison of A1C EZ 2.0 *vs.* Bio-Rad VARIANT-II**			
	**Passing-Bablok regression equation,** **y = a (95% CI) + b (95% CI) x**	**Mean difference** **(limits of agreement)**	**Mean relative difference, %** **(limits of agreement)**
All subjects	y = 0.03 (- 0.14 to 0.20) + 1.02 (1.00 to 1.05) x	0.17 (- 0.52 - 0.85)	2.5 (- 7.3 - 12.4)
SGI (venous group)	y = 0.30 (- 0.12 to 0.30) + 1.00 (1.00 to 1.06) x	0.24 (- 0.41 - 0.89)	3.4 (- 5.4 - 12.2)
SGII (capillary group)	y = - 0.36 (- 0.65 to 0.10) + 1.06 (1.00 to 1.10) x	0.06 (- 0.64 - 0.75)	0.7 (- 8.5 - 10.0)
SGIII (for venous)	y = - 0.10 (- 0.39 to 0.30) + 1.06 (1.00 to 1.10) x	0.24 (- 0.36 - 0.83)	3.8 (- 5.1 - 12.7)
SGIII (for capillary)	y = 0.20 (- 0.33 to 0.20) + 1.00 (1.00 to 1.09) x	0.16 (- 0.58 - 0.90)	2.7 (-8.9 - 14.2)
**Comparison of A1C EZ 2.0 *vs.* Tosoh HLC-723 G8**			
All subjects	y = - 0.12 (- 0.27 to 0.00) + 1.02 (1.00 to 1.05) x	0.04 (- 0.57 - 0.66)	0.6 (- 8.0 - 9.2)
SGI (venous group)	y = 0.10 (0.04 to 0.10) + 1.00 (1.00 to 1.02) x	0.09 (- 0.51 - 0.69)	1.3 (- 6.6 - 9.3)
SGII (capillary group)	y = - 0.46 (- 0.76 to 0.00) + 1.06 (1.00 to 1.11) x	- 0.04 (- 0.72 - 0.65)	- 0.6 (- 9.6 - 8.4)
SGIII (for venous)	y = - 0.32 (0.59 to 0.10) + 1.06 (1.00 to 1.10) x	0.08 (- 0.44 - 0.60)	1.1 (- 6.8 - 9.0)
SGIII (for capillary)	y = - 0.20 (0.52 to 0.00) + 1.04 (1.00 to 1.09) x	0.05 (- 0.52 - 0.63)	0.8 (- 8.1 - 9.7)
**Comparison of A1C EZ 2.0 *vs.* Tosoh HLC-723 G8 Premier HB9210**			
All subjects	y = 0.00 (- 0.00 to 0.00) + 1.00 (1.00 to 1.00) x	0.02 (- 0.57 - 0.61)	0.4 (- 7.3 - 8.2)
SGI (venous group)	y = 0.22 (0.00 to 0.43) + 0.97 (0.94 to 1.00) x	- 0.05 (- 0.67 - 0.56)	- 0.4 (- 8.0 - 7.2)
SGII (capillary group)	y = 0.00 (- 0.25 to 0.00) + 1.00 (1.00 to 1.04)x	0.00 (- 0.64 - 0.65)	0.2 (- 7.8 - 8.2)
SGIII (for venous)	y = - 0.29 (- 0.54 to - 0.10) + 1.06 (1.00 to 1.10) x	0.11 (- 0.34 - 0.56)	1.6 (- 5.4 - 8.6)
SGIII (for capillary)	y = - 0.37 (- 0.64 to - 0.11) + 1.07 (1.02 to 1.11) x	0.07 (- 0.44 - 0.58)	1.0 (- 6.7 - 8.8)
**Comparison of A1C EZ 2.0 *vs.* mean SRM**			
All subjects	y = 0.10 (- 0.17 to 0.10) + 1.00 (1.00 to 1.04) x	0.08 (- 0.50 - 0.65)	1.1 (- 6.9 - 9.2)
SGI (venous group)	y = 0.10 (0.10 to 0.10) + 1.00 (1.00 to 1.00) x	0.09 (- 0.48 - 0.67)	1.4 (- 6.1 - 9.0)
SGII (capillary group)	y = - 0.30 (- 0.56 to 0.00) + 1.05 (1.00 to 1.08) x	0.01 (- 0.63 - 0.64)	0.1 (- 8.2 - 8.4)
SGIII (for venous)	y = - 0.22 (- 0.49 to 0.10)+ 1.06 (1.00 to 1.10) x	0.14 (- 0.33 - 0.61)	2.1 (- 5.1 - 9.4)
SGIII (for capillary)	y = - 0.23 (- 0.54 to 0.10) + 1.05 (1.00 to 1.10) x	0.09 (- 0.44 - 0.63)	1.4 (- 7.0 - 9.8)

### Bias analysis

**Figure 3:**
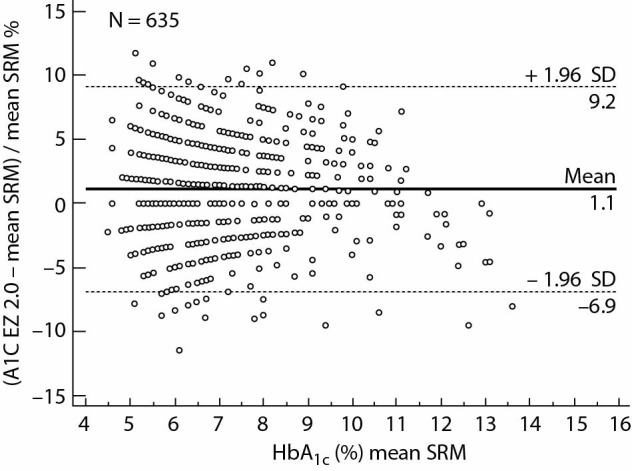
Bland-Altman relative difference plot shows the differences between the paired A1C EZ 2.0 results and the mean SRM. Horizontal lines are drawn at the mean difference% (solid line) and at the limits of agreement (dashed lines). Mean relative difference of A1C EZ 2.0 results *versus* the mean SRM is 1.1%, with the limits of agreement from -6.9% to 9.2%. SRM- secondary reference method.

The mean difference between A1C EZ 2.0 values *versus* mean SRM is 0.08% HbA_1c_ (mean relative difference of 1.1%) (Figure 3 and Table 2). Respective mean difference for each reference HPLC device was 0.17% HbA_1c_ for Bio-Rad Variant II Turbo, 0.04% HbA_1c_ for Tosoh HLC-723 G8 and 0.02% HbA_1c_ for Premier Hb9210 (mean relative difference of 2.5%, 0.6% and 0.4%, respectively). All detailed statistics for each subject group are listed in Table 2. Bland-Altman difference plot of A1C EZ 2.0 results *vs* mean SRM shows that 96.5% of results have relative differences from - 6.9% to 9.2% (Figure 3). In addition, 84.6% of data has ARD lower than 6%, and 94.2% of data has ARD lower than 8%. MARD of 3.26% of A1C EZ 2.0 results compared with mean SRM was found.

### Differences in HbA_1c_ values depending upon types of blood sample

In this study, no notable difference in HbA_1c_ values between venous and fingertip capillary whole blood of SGIII subjects was seen as measured by A1C EZ 2.0. Passing-Bablok regression analysis towards paired data from venous and fingertip capillary whole blood yielded the equation y = - 0.10 + 1.00 x; with 95%CI of intercept - 0.10 to 0.08, and slope: 0.98 to 1.00, which indicates that there is no significant difference between HbA_1c_ values on measurement with different kinds of blood sample. Also, there was no significant difference in HbA_1c_ values between venous and capillary blood on assessment with reference HPLC devices (Bio-Rad Variant II Turbo: y = 0.00 (0.00 to 0.00) + 1.00 (1.00 to 1.00) x; Tosoh HLC-723 G8: y = 0.0 (0.00 to 0.00) + 1.0 (1.00 to 1.00) x; Premier Hb9210: y = 0.0 (0.00 to 0.00) + 1.00 (1.00 to 1.00) x.

### Precision

A1C EZ 2.0 showed good precision in the twenty days evaluation period (Table 3). The total CV is 2.3% for low HbA_1c_ sample (5.2% HbA_1c_) and 1.9% for high HbA_1c_ sample (11.6% HbA_1c_). In addition, the within-run CV, between-run CV and between-day CV all fell in the acceptable region (less than 2%, Table 3), implying that A1C EZ 2.0 has consistent performance over a certain period of time under different circumstances.

**Table 3:** Coefficients of variation calculated according to CLSI EP5-A2

**CV (%)**	**Sample 1** **(HbA_1c_ = 5.2%)**	**Sample 2** **(HbA_1c_ = 11.6%)**
Within-run	1.9%	1.8%
Between-run	0.0%	0.6%
Between-day	1.4%	0.0%
Total	2.3%	1.9%
CV – coefficient of variation.		

### Sensitivity and specificity of test by A1C EZ 2.0

We further evaluated the sensitivity and specificity of A1C EZ 2.0. While considering Premier Hb9210 results as reference and HbA_1c_ of 6.5% as clinical decision level for DM diagnosis, the sensitivity and specificity of A1C EZ 2.0 were both 96%. When mean SRM was used as reference, sensitivity and specificity of A1C EZ 2.0 were 96% and 93%, respectively (Table 4).

**Table 4:** Diagnostic sensitivity and specificity of A1C EZ 2.0 for diagnosing of diabetes

**HbA1c cut-off value**		**A1C EZ 2.0 *vs.* Bio-Rad VARIANT-II**	**A1C EZ 2.0 *vs.*** **Tosoh HLC-723 G8**	**A1C EZ 2.0 *vs.* Premier HB9210**	**A1C EZ 2.0 *vs.*** **mean SRM**
6.5%	Sensitivity (%)	96	95	96	96
	Specificity (%)	90	96	96	93
6.3%	Sensitivity (%)	97	95	96	97
	Specificity (%)	87	92	89	89
SRM- secondary reference method.					

## Discussion

A large scale clinical study in the hospital setting was designed to evaluate the performance of a novel POC HbA_1c_ device A1C EZ 2.0. Overall performance of A1C EZ 2.0 was fairly good in our study, and was similar to the previously reported performance by another study (*7*). What is different is that in this study we used the clinical specimens including the fingertip capillary whole blood, venous blood samples and both venous blood samples and fingertip capillary whole blood samples from the same participant. Analysis of our results indicates that A1C EZ 2.0 has smallest mean difference (0.02% HbA_1c_) with Premier Hb9210, and slightly less consistence with Tosoh HLC-723 G8 and Bio-Rad Variant II Turbo. This might be as both A1C EZ 2.0 and Premier Hb9210 use boronate affinity method. Boronate affinity method was proven to have good tolerance to some common interference factors such as haemoglobin variants and certain drugs, which usually affect the HbA_1c_ results determined by cation-exchange method (*13*, *14*). In this study, sample from one patient (data was excluded in our statistics) was found to have haemoglobin abnormality and both Bio-Rad Variant II Turbo and Tosoh HLC-723 G8 failed to give reasonable results, while Premier Hb9210 and A1C EZ 2.0 gave similar HbA_1c_ value (7.6% and 7.8% HbA_1c_, respectively). For several other samples where the relative differences between Premier Hb9210 and Tosoh HLC-723 G8 or Bio-Rad Variant II Turbo were more than 10%, we inferred that some interference factors might have existed in those samples and Premier Hb9210 results were considered to be more reliable.

Although venous blood may differ from capillary blood to some extent, in many aspects, such as total protein, blood cell concentration and ion concentration, the HbA_1c_ value usually shows no notable difference when measured using different type of blood sample in different instruments (*15*-*19*). There was no significant difference in HbA_1c_ values between venous blood and fingertip capillary whole blood of SGIII subjects as seen on measurement by A1C EZ 2.0.

Sensitivity and specificity analysis of A1C EZ 2.0 indicates that the risk of false positive and negative results is very low with evaluation by A1C EZ 2.0. Several former studies have shown that the ideal cut-off HbA_1c_ values for diagnosis of DM varies among ethnic groups, and the best clinical decision level for Chinese population might be 6.3% (*20*-*23*). We also used 6.3% as cut-off value, and found that the sensitivity and specificity of A1C EZ 2.0 was 96% and 89% as compared to Premier Hb9210 as reference, and 97% and 89% as compared to mean SRM as reference. In this analysis, when clinical decision level was changed from 6.5% to 6.3%, high sensitivity was maintained, whereas specificity dropped as compared to both references (Table 4). In conclusion, the sensitivity and specificity of A1C EZ 2.0 meets the general clinical requirement for HbA_1c_ testing, and can be used for clinical diabetes diagnosis.

As the POC HbA_1c_ devices can provide results in short time and at relatively lower cost, these can help in improving the doctor-patient interaction, appropriate modification of the treatment if required and/or diagnosis/screening of new cases of DM from large population with higher efficiency. On the other hand, doubts towards POC HbA_1c_ devices have been raised in some studies showing that some POC HbA_1c_ devices may not be suitable for clinical use due to their unsatisfactory precision and/or variation in different lots (*5*, *6*). Thus, more precise and accurate POC HbA_1c_ device is in urgent need, especially in countries like China, with an increasing incidence and prevalence of DM, as expensive HPLC HbA_1c_ instruments are usually inaccessible and unaffordable to many grassroots medical institutions.

While it is widely accepted that HPLC devices represent one of the most accurate way to measure HbA_1c_, we found that performance of POC HbA_1c_ device is not significantly different from the HPLC devices in this study. The 84.7% of Bio-Rad Variant II Turbo results have ARD lower than 6%, and 94.6% have lower than 8% as compared with Premier Hb9210 results. Both values are slightly lower than A1C EZ 2.0 87.6% and 96.5%, respectively (data not shown). The controls were tested before and after every day’s experiments and routine calibrations were carried out in all HPLC devices to confirm that performance of all instruments were in the claimed acceptable region by manufacturers. However, Bio-Rad Variant II Turbo results show consistent negative bias (about - 0.2% HbA_1c_) as compared with Tosoh HLC-723 G8, Premier Hb9210, A1C EZ 2.0 in our study. This negative bias was observed in all three subject groups and through seven months period of this study. Such kind of bias might have occurred due to the change of condition of cation-exchange column during use, or the individual calibration process we performed, as both are commonly seen in clinical applications. On the other hand, use of POC HbA_1c_ device like A1C EZ 2.0, which usually uses single cartridge or strip for one test can decrease the risk of systematic bias and might achieve similar performance as HPLC in clinical practice while having slightly higher CV than HPLC device at the same time.

In conclusion, we verified a novel POC HbA_1c_ device A1C EZ 2.0, and found its performance met the general clinical requirement in this study. However, there are still some limitations in our study. Only one lot of strip of A1C EZ 2.0 was used in this study, thus lot-to-lot variation of strips has not been evaluated; we also did not evaluate the stability of test strip of A1C EZ 2.0 in this work. Tests by A1C EZ 2.0 and HPLC instruments were not performed at exactly the same point of time. All these limitations merit further investigation in future.
